# Treatment patterns and persistence on disease modifying therapies for multiple sclerosis and its associated factors

**DOI:** 10.1186/s12883-024-03594-3

**Published:** 2024-04-02

**Authors:** Simón Cárdenas-Robledo, Laura Estefanía Arenas-Vargas, Rubén Darío Arenas, Jorge Mario Gaspar-Toro, Ángela María Muñoz-Rosero, Aranza Helena Tafur-Borrero, Daniel Stiven Marín-Medina, Hernan Andrés Acosta-Fajardo, Claudia Guío-Sánchez, Lorena López-Reyes

**Affiliations:** 1 Centro de Esclerosis Múltiple (CEMHUN), Departamento de Neurología, Hospital Universitario Nacional de Colombia, Calle 44 # 59-75, Bogotá, Colombia; 2 Departamento de Medicina Interna, Facultad de Medicina, Universidad Nacional de Colombia, Bogotá, Colombia

**Keywords:** Multiple sclerosis, Disease modifying therapy, Network analysis, Treatment adherence, Persistence on treatment, Treatment patterns

## Abstract

**Background:**

Effective interventions for Multiple Sclerosis require timely treatment optimization which usually involves switching disease modifying therapies. The patterns of prescription and the reasons for changing treatment in people with MS, especially in low prevalence populations, are unknown.

**Objectives:**

To describe the persistence, reasons of DMT switches and prescription patterns in a cohort of Colombian people with MS.

**Methods:**

We conducted a retrospective observational study including patients with confirmed MS with at least one visit at our centre. We estimated the overall incidence rate of medication changes and assessed the persistence on medication with Kaplan–Meier survival estimates for individual medications and according to efficacy and mode of administration. The factors associated with changing medications were assessed using adjusted Cox proportional-hazards models. The reasons for switching medication changes were described, and the prescription patterns were assessed using network analysis, with measures of centrality.

**Results:**

Seven hundred one patients with MS were included. Mean age was 44.3 years, and 67.9% were female. Mean disease duration was 11.3 years and 84.5% had relapsing MS at onset, with median EDSS of 1.0. Treatment was started in 659 (94%) of the patients after a mean of 3 years after MS symptom onset. Among them, 39.5% maintained their initial DMT, 29.9% experienced a single DMT change, while 18.7% went through two, and 11.9% had three or more DMT changes until the final follow-up. The total number of treatment modifications reached 720, resulting in an incidence rate of 1.09 (95% confidence interval: 1.01–1.17) per patient per year The median time to change after the first DMT was 3.75 years, and was not different according to the mode of administration or efficacy classification. The main reasons for changing DMT were MS activity (relapses, 56.7%; MRI activity, 18.6%), followed by non-serious adverse events (15.3%) and disability (11.1%). Younger age at MS onset, care under our centre and insurer status were the main determinants of treatment change. Network analysis showed that interferons and fingolimod were the most influential DMTs.

**Conclusions:**

A majority of patients switch medications, mostly due to disease activity, and in association with age and insurer status.

**Supplementary Information:**

The online version contains supplementary material available at 10.1186/s12883-024-03594-3.

## Introduction

Multiple Sclerosis (MS) is a chronic condition that affects more than 2.8 million individuals around the world and is a leading cause of non-traumatic disability, associated with substantial morbidity, healthcare resource use and overall economic burden [1]. The therapeutic scenario for MS has changed considerably over the last 20 years [2], due to the advent of a wide range of disease modifying therapies (DMTs) with different mechanisms of action, efficacy and safety profile [3].

Many DMTs are currently approved by the U.S. Food and Drug Administration (FDA) and the European Medicines Agency (EMA); however, most of them are approved for relapsing MS and only ocrelizumab (for primary progressive MS) and siponimod (for active secondary progressive MS) are so for progressive forms of MS. Despite this, it is now possible to base the initial selection of DMTs on clinical characteristics and prognostic factors of each individual case, as well as the presence of comorbidities and individual preferences regarding efficacy and safety [4]. The availability of DMTs has also allowed for switching between DMT in response to breakthrough disease, tolerability and safety issues [5].

The frequency of MS is increasing worldwide, including in Latin-American countries, where the access to MS care is limited and heterogeneous [6]. DMTs impose an important economic burden on the healthcare systems, accounting for more than two-thirds of the total direct cost of MS care [7]. The economic impact of MS in the healthcare system of Colombia has been scarcely studied [8, 9], and the behaviour of DMTs prescription is unknown. Therefore, the study of prescription patterns of DMTs is important both from the patient as well as from the payer’s perspective.

In this regard, the aim of this study was to ascertain the persistence on DMTs, investigate the frequency, reasons and determinants for switching between DMTs, and describe their prescription patterns in a real-life scenario in our country.

## Methods

### Setting, design and population

This is an observational study performed in a single centre in Bogotá, Colombia.

We included all people with MS (pwMS) confirmed according to the 2017 revisions of the McDonald criteria [10], who had had at least one visit in our centre between May 2016 through December 2020. Subjects were excluded if they had incomplete records regarding the basic clinical and demographic variables. The inclusion/exclusion criteria here described are those approved by the ethics committee.

### Variables and data collection

Records were retrospectively reviewed between September and December 2020 and data were gathered using Redcap [11]. Basic demographic (age, sex) as well as clinical variables such as age and MS phenotype at onset [12], disease duration, the disability assessment in the last evaluation (using the expanded disability status scale EDSS [13]) and age at treatment onset were collected. For each DMT started, the date of the first and last dose was documented. If these dates were unknown, they were imputed according to a pre-specified protocol: when only the year was known, a central date was assigned, specifically the 15th of June in the respective year. When both the month and year were known, we assigned the 15th of the known month and the respective year.

The main outcome was a change in the DMT used and was defined as the moment when a subsequent DMT was started. When the exact date of the post-change DMT first dose was unknown, the moment of the DMT change was defined as the moment of the last dose of the pre-change DMT. DMTs were grouped by mode of administration (oral, self-injectables, IV), and high (natalizumab, alemtuzumab, ocrelizumab, rituximab and fingolimod) vs. low efficacy (interferons, glatiramer acetate, dimethyl fumarate and teriflunomide). The latter classification was done in accordance with our institutional protocol. Cladribine, ofatumumab and most sphingosine 1-phosphate receptor inhibitors (except for fingolimod) were not available in our country at the time of the study and are therefore not included in the analysis. The reasons for the treatment changes were categorised as 1) disease activity: relapses, disability worsening or MRI activity, as defined by the treating physician at the moment; 2) safety: serious (leukopenia, anaemia, thrombocytopenia, liver injury, skin necrosis, macular oedema, any opportunistic and/or recurring infection, hypersensitivity reactions, systemic autoimmunity, any neoplasm) and non-serious (any other) adverse events; 3) reproductive issues: planning to get pregnant, unexpected pregnancy, breastfeeding; and 4) administrative issues, such as insurance coverage/dispensation or changes in insurances. If the reason for DMT changes was unknown, it was reported as such.

Suspension of DMTs was defined as the interruption of treatment that was not resumed with the same or a different medication within six months. For anti-CD20 monoclonal antibodies, this period was considered to be one year. For alemtuzumab persistence was defined as the time between the first dose of the index medication and the first dose of a subsequent DMT.

### Ethics

This study was conducted in accordance with the declaration of Helsinki and approved by the local ethics committee (Comité Ética de la Investigación, Hospital Universitario Nacional de Colombia, project ID: CEI-2020-07-02), with a waiver for informed consent.

### Statistical analysis

Continuous variables were described with central tendency (mean/median) and dispersion (standard deviation -SD- or interquartile range -IQR-) as appropriate from their distribution. Categorical variables were described in terms of absolute and relative frequencies.

The frequency of treatment changes was assessed by estimating the annual incidence with 95% confidence intervals (95%CI). The reasons for treatment change were described for each DMT.

We estimated the overall persistence on DMTs and cumulative hazard of treatment switches over time using Kaplan–Meier estimates assuming the changes were recurrent in nature. Persistence on DMTs was also analysed by mode of administration and high vs. low efficacy. We also assessed the factors associated with the risk of treatment changes in general with hazard ratios (HR) calculated from multivariate models, using the Prentice, Williams and Peterson-gap time version of the Cox proportional hazard model [14, 15]. For these we included baseline variables (sex, age at MS onset, phenotype at MS onset, insurer) and whether each treatment change occurred under the care of our specialised centre. Age and phenotype at MS onset were included in the models because of evidence relating higher onset age with long term disability [16], and the differences in the number of approved DMTs for relapsing and progressive forms of MS, both of which might drive the decisions to change treatment. These might also be influenced by the degree of specialisation of the physician or multidisciplinary team caring for the patient, which was the reason for including whether the treatment changes were done under our specialised care. Given that longer disease duration potentially confers a higher likelihood of treatment changes over time, we included this variable in the models as well. The models included the reasons for each treatment change (classified as disease activity, safety and others), in order to assess if the baseline variables were independently associated with the risk of treatment switches. The proportional hazards assumption of the model was assessed using the Schoenfeld’s test.

Finally, the patterns of DMT prescription were evaluated using network analysis after creating an adjacency matrix that described the relationships between pre-and post-change DMTs. The resulting network was described graphically and analysed with different measures of vertex importance. These included 1) measures of degree: how many connections (both to and from) have the different DMTs in the network; 2) eigenvector centrality: a measure of how DMTs are connected to other DMTs that are highly interconnected in the network; 3) betweenness: how frequently a DMT lies on the shortest path(s) between any two DMTs in the network; and 4) closeness: how short the shortest paths are from a DMT to all other DMTs.

Statistical analysis was done using R, version 4.0.2 [17]. This report is compliant with the Strengthening the Reporting of Observational studies in Epidemiology (STROBE) guidelines (Supplementary Table S1) [18].

### Bias and study size

As this is a retrospective study based on historical records it is subject to a strong recall bias. In order to mitigate it at least for the basic demographic and clinical variables, we have developed a standard registration format in our electronic medical records. We did not perform an *a* *priori* sample size calculation. Given that the necessary information was easily accessible, we aimed to assess the census of our cohort. For the multivariable analysis, our sample exceeds the recommended 20 events per variable included [19].

## Results

### Participants and descriptive data

After the exclusion of 54 subjects due to incomplete records, a total of 701 patients were included in the study. 67.9% were women, with mean (SD) age of 44.3 (12.1) years. Most (84.5%) pwMS had a relapsing onset, with median (IQR) EDSS in the last evaluation of 1.0 (3.5). Mean (SD) follow-up between treatment onset and last evaluation was 6.4 (9.1) years. Treatment was started in 659 (94%) of the pwMS after a mean (SD) of 3.0 (5.1) years after MS onset. The DMTs most commonly used first were interferons (57.8%), followed by fingolimod (15.2%). Other clinical and demographic variables are described in Table 1. Among the 54 individuals excluded from the study five (9.25%) had started treatment DMT, but their inclusion was hindered because of the missing information on the date of treatment start. The remaining 49 had not started treatment and were excluded due to insufficient clarity in diagnostic information. For 100% of the DMTs, we had information on the year of initiation. The month and year were known for nearly half of these cases. The exact date of DMT onset was known for 10% of the cases.

**Table 1:** Basic demographic and clinical characteristics of a sample of included pwMS

		***N = 701***
Age *years, mean (SD)*		44.3 (12.1)
Sex *female, n (%)*		476 (67.9)
Onset phenotype* relapsing, n (%)*		592 (84.5)
Age at MS onset *years, mean (SD)*		32.8 (10.7)
EDSS in the last evaluation *n (%)*		
< 3.0		501 (71.5)
3.5—6.0		140 (20.0)
> 6.0		60 (8.6)
Disease duration *years, mean (SD)*		11.3 (8.2)
Diagnostic delay^a^ * years, mean (SD)*		2.2 (4.5)
Treatment delay^b^ * years, mean (SD)*		3.0 (5.1)
Time from diagnosis to treatment *years, mean (SD)*		0.9 (3.5)
Follow-up^c^ * years, mean (SD)*		6.4 (9.1)

*EDSS* Expanded Disability Status Scale, *MS* Multiple Sclerosis, *SD* Standard deviation

^a^Time from MS onset to diagnosis confirmation

^b^Time from MS onset to treatment onset

^c^Time from treatment onset to last evaluation

### Treatment changes

In pwMS who started treatment at any moment, we found a total of 710 treatment changes: 404 (56.9%) were switches between DMTs, 186 (26.2%) were treatment suspensions and 120 (16.9%) were treatment resumptions. The overall incidence (95%CI) of any treatment change was 1.09 (1.01–1.17) per patient-years. The reasons identified for such changes were disease activity in 71.8%, adverse events in 16.2%, administrative issues in 6.7% and reproductive aspects in 5.3%. The reasons for treatment switch and suspension of individual DMTs are described in Table 2.

**Table 2:** Reasons for treatment changes according to each individual DMT

	**Alemtuzumab**	**Dimethylfumarate**	**Fingolimod**	**Glatiramer**	**Interferons**	**Natalizumab**	**Rituximab**	**Teriflunomide**
	**Treatment switch** ** (** ***N*** ** = 404)**							
**Total**	3	7	79	22	219	47	4	23
**Disease activity** *n (%)*	3 (100)	3 (42.9)	62 (77.2)	9 (40.1)	116 (52.9)	11 (23.4)	2 (50.0)	14 (60.8)
**Safety** *n (%)*	0 (0.0)	2 (28.6)	11 (13.9)	4 (18.2)	69 (31.5)	4 (8.5)	1 (25.0)	6 (26.1)
**Reproductive issues** *n (%)*	0 (0.0)	0 (0.0)	1 (1.3)	0 (0.0)	0 (0.0)	0 (0.0)	0 (0.0)	0 (0.0)
**Administrative issues** *n (%)*	0 (0.0)	0 (0.0)	0 (0.0)	2 (9.5)	6 (2.8)	1 (2.1)	0 (0.0)	0 (0.0)
**JC virus** *n (%)*	0 (0.0)	0 (0.0)	0 (0.0)	0 (0.0)	0 (0.0)	33 (70.2)	0 (0.0)	0 (0.0)
**Other** *n (%)*	0 (0.0)	0 (0.0)	3 (3.8)	2 (9.5)	7 (3.2)	1 (2.1)	1 (25.0)	1 (4.3)
**Unknown** *n (%)*	0 (0.0)	2 (28.6)	3 (3.8)	4 (18.2)	33 (15.1)	2 (4.3)	0 (0.0)	2 (8.7)
	**Treatment suspension** ** (** ***N*** ** = 186)**							
**Total**	2	5	21	11	106	24	9	8
**Disease activity** *n (%)*	0 (0.0)	0 (0.0)	1 (4.8)	0 (0.0)	15 (14.2)	1 (4.2)	2 (25.0)	0 (0.0)
**Safety** *n (%)*	0 (0.0)	2 (40.0)	3 (14.3)	2 (18.2)	27 (25.5)	0 (0.0)	1 (12.5)	1 (12.5)
**Reproductive issues** *n (%)*	0 (0.0)	1 (20.0)	6 (28.6)	4 (36.4)	17 (16.0)	4 (16.7)	0 (0.0)	2 (25.0)
**Administrative issues** *n (%)*	0 (0.0)	2 (40.0)	9 (42.9)	2 (18.2)	13 (12.3)	9 (37.5)	4 (50.0)	4 (50.0)
**JC virus** *n (%)*	0 (0.0)	0 (0.0)	0 (0.0)	0 (0.0)	0 (0.0)	11 (45.8)	0 (0.0)	0 (0.0)
**Other** *n (%)*	2 (100.0)	0 (0.0)	1 (4.8)	2 (18.2)	14 (13.2)	1 (4.2)	0 (0.0)	1 (12.5)
**Unknown** *n (%)*	0 (0.0)	0 (0.0)	2 (9.5)	2 (18.2)	27 (25.5)	2 (8.3)	1 (12.5)	1 (12.5)

**Fig. 1:**
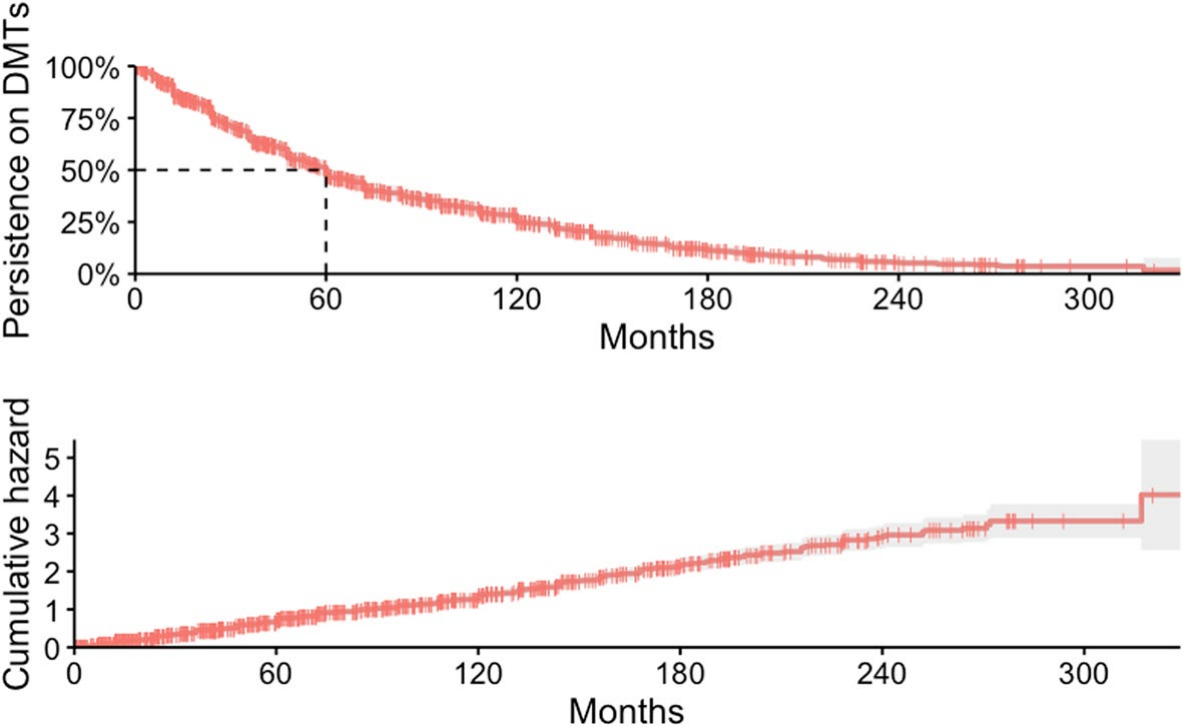
Persistence on DMTs in general (top panel) and cumulative hazard of any treatment switch over time (bottom panel). Dashed line shows the median persistence on DMTs (see results for details)

Treatment changes in general occurred at a median (95%CI) of 60.0 (54.3–63.0) months after treatment onset (Fig. 1). The median time from treatment onset to switch for individual DMTs was for interferons (61.3 months; 95%CI 60.0–72.0), glatiramer (31 months; 95%CI 18.0–53.0), fingolimod (70.0 months; 95%CI 61.8–96.0), and natalizumab (45.0 months; 95%CI 36.0–67.3). For the remaining DMTs either median time or 95%CIs were not estimable due to low numbers of treated subjects or events (Supplementary Fig. S1).

After suspension, treatment was resumed after a median of 31 months (95%CI 24.0–41.8). The reasons for such suspensions are described in Table 2.

**Fig. 2:**
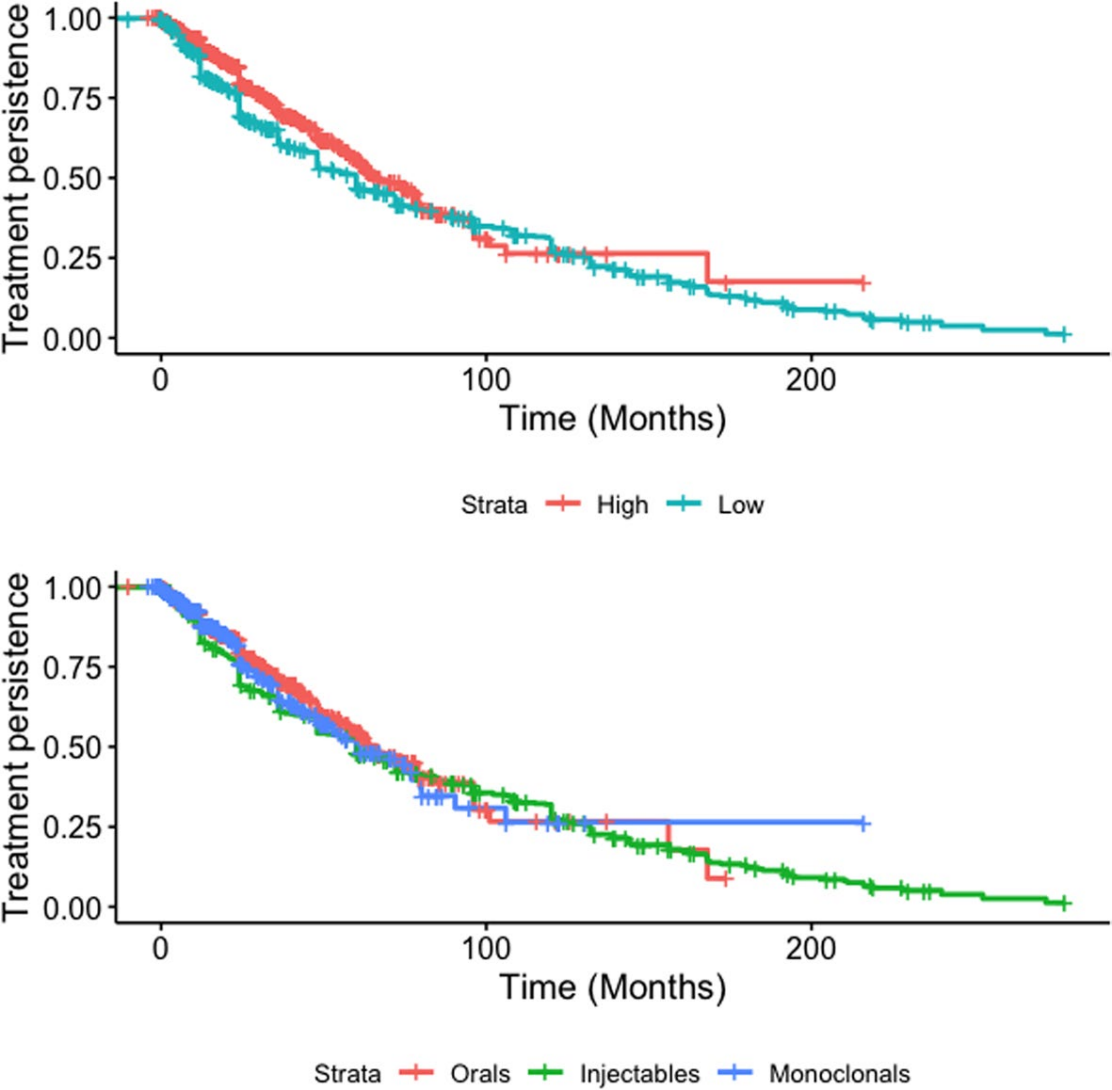
Persistence on DMTs according to their efficacy classification (top panel) and mode of administration (bottom panel)

Treatment persistence was very similar while using injectables (60.0 months; 95%CI 48–72), orals (65.1 months; 95%CI 58.0–79.0) or monoclonal antibodies (60.0 months; 95%CI 48.3–80.1), as was for high (66.8 months; 95%CI 61.0–79.0) and low efficacy DMTs (60.0 months; 95%CI 48.0–67.1) (Fig. 2).

After adjusting for covariates and for the reasons for switching, the risk of treatment changes was found to be significantly increased when pwMS were cared for at our centre and decreased with increasing age at onset of MS and according to the insurer status (Table 3).

**Table 3:** Association of treatment changes with clinical and demographic variables according to univariable and multivariable Cox proportional hazard models

	Univariable			Multivariable		
	HR	95% CI	*p*-value	HR	95% CI	*p*-value
**Sex** ^a^	0.98	0.84–1.15	0.8	0.81	0.68–0.96	0.017
**Phenotype** ^b^	1.04	0.87–1.24	0.7	1.17	0.96–1.42	0.13
**Disease duration** ^c^	0.96	0.95–0.97	< 0.001	0.94	0.93–0.95	< 0.001
**Age at MS onset** ^d^	0.99	0.98–1.00	0.004	0.99	0.98–0.99	< 0.001
**Insurer** ^e^	0.71	0.61–0.83	< 0.001	0.67	0.57–0.79	< 0.001
**HUN** ^f^	2.50	2.13–2.92	< 0.001	1.77	1.49–2.10	< 0.001
**Diagnostic delay** ^g^	0.97	0.95–1.00	0.015	0.99	0.97–1.01	0.5

*CI* Confidence Interval, *EDSS* Expanded Disability Status Scale, *HUN* Hospital Universitario Nacional, *HR* Hazard Ratio, *MS* Multiple Sclerosis

^a^Male as reference

^b^Relapsing compared to progressive course at onset

^c^Time between symptom onset and last visit, per one year

^d^Change in risk per each year

^e^The insurer with the least pwMS included as reference

^f^Whether the treatment change was done under the care at our centre

^g^Time between symptom onset and diagnosis, per one year

Schoenfeld’s test showed that the proportional hazards assumption was held for all variables except for disease duration and switching treatment under our care (Supplementary Table S2).

### Treatment patterns

**Fig. 3:**
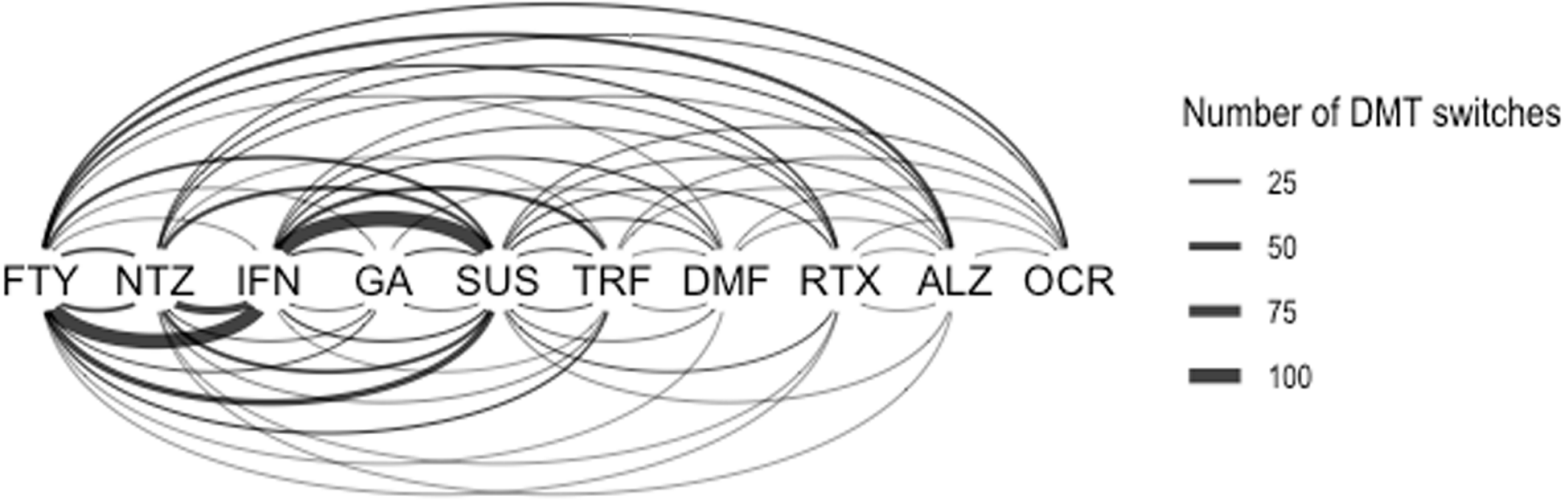
Network analysis of the treatment patterns. Lines on the top of the nodes depict switches from left to right and lines below the nodes depict switches from right to left. FTY: fingolimod; NTZ: natalizumab; IFN: interferons; GA: glatiramer acetate; SUS: suspension; TRF: teriflunomide; DMF: dimethylfumarate; RTX: rituximab; ALZ: alemtuzumab: OCR: ocrelizumab

The matrix of relationships showed that most DMT switches occurred between interferons and suspension and from interferons to fingolimod (Supplementary Table S3). The measures of DMT importance within the network showed that the most central DMTs were the interferons (eigenvector centrality: 1.0; betweenness: 0.0; closeness: 0.019), followed by the status of suspension (eigenvector centrality: 0.888; betweenness: 0.0; closeness: 0.016). The most marginal DMT was ocrelizumab (eigenvector centrality: 0.097) (Fig. 3 and Supplementary Table S4).

## Discussion

In this observational study we describe the frequency, reasons and determinants of switching or suspension of DMTs, as well as the patterns of DMT prescription in our cohort. Our results show that most pwMS who started treatment had a DMT switch at some point, with disease activity being the most common reason for change, and that the persistence on treatment was similar for the different DMTs, regardless of their efficacy or mode of administration. In addition, the insurer status of the pwMS was found to be the main determinant of DMT switching. Most treatment switches involved interferons fingolimod and treatment suspension.

Our findings are in line with similar previous studies, which show that most pwMS treated will eventually change their therapy [20–23]. However, the treatment persistence in different studies varies, with median time to treatment switch ranging from 25 months [22] to 50 months [20, 21]. In our study, treatment persistence was longer, with a median time of 60 months. The differences in these results are still to be addressed, but the structure of the healthcare system and the availability of different DMTs in different countries might be determinant. Other factors that influence the persistence on DMTs, such as comorbidities [23, 24] might contribute to the differences in the results. However, our study did not assess the presence of comorbidities, which is a limitation that needs to be acknowledged.

We find it interesting that the treatment persistence was very similar across the different DMTs in terms of their classification based on efficacy and mode of administration. This leads to thinking that the decision to switch or stop therapy might be driven by factors different from those, such as cost, and continuous availability of the medications involved.

The most common reason for treatment change was disease activity. This is in line with previous findings [25, 26], although in these studies a minority of pwMS treated with high-efficacy DMTs were assessed.

In our study, age at MS onset and insurer status and the care at our centre were the only variables associated with the risk of switching therapies even after considering the reason for treatment change. The former has several interpretations. It is possible that pwMS with younger age at onset had a more active disease than pwMS with onset at an older age, which might increase the likelihood of treatment switching due to lack of efficacy. Similar findings were reported by previous studies, which described that subjects who were younger when starting a DMT, were more likely to discontinue therapy [20, 21, 23]. On the other hand, pwMS with MS onset at older age are more likely to have progressive MS, for which the therapeutic options are limited, leading to a decreased likelihood of treatment switches, so these results should be interpreted carefully. It is important that more than half of the pwMS received interferons as the first treatment, and thus the overall risk of treatment changes might be driven by the behaviour of treatment switches from this group of medications. Although these first line medications have a mild efficacy to prevent relapses, and therefore would be expected to have been promptly switched, it is important to bear in mind that they were the only medications available for nearly 10 years. The use of other first line DMTs such as dimethylfumarate is low in our study, possibly due to the fact it was approved in our country rather recently (late 2016), well after other DMTs. The prescription behaviour is likely to have changed in the last couple of years, which is an important issue for future research.

pwMS with younger age at onset and enough disease duration to date were probably treated during times in which no alternatives to interferons were available. On the other hand, young pwMS with recent MS onset might not have had enough follow-up time to need a treatment switch, or might have been treated earlier with higher efficacy DMTs, and therefore might not have had disease activity or safety issues that prompted the change of treatment.

Disability has been previously reported to be associated with treatment persistence [23, 26]. Our study was not able to confirm this in our sample due the impossibility to assess disability in retrospect, particularly for those treatment changes occurring before the care at our centre. This is another limitation of the retrospective design of our study.

Being suboptimal treatment response the most common reason for switching DMTs, it will be interesting to assess the switching behaviour in the future, when more pwMS are exposed to DMTs with higher efficacy, earlier in the course of the disease, and with long-lasting immunological effects (such as alemtuzumab, cladribine and anti-CD20 monoclonal antibodies).

The analysis of treatment patterns revealed that most switches occurred around the interferons, fingolimod and treatment suspension. This is likely explained due to the long time the interferons and fingolimod were the only available therapeutic options in our country, but also the high frequency of tolerability issues associated with the use of interferons.

The most relevant finding in our opinion is the influence of the insurer in the risk of treatment changes. Healthcare provision in our country relies on public funding that is administered by private insurers. Mandatory affiliation to chosen insurers applies to employed individuals and those with independent economic activities, along with their families. Insurers are responsible for providing care directly or by outsourcing to third parties for diagnostic tests, consultations, admissions, or medications, at their discretion. Individuals lacking the financial means to participate in the contributive system are covered by a subsidised branch of the healthcare system. Combined, these branches extend healthcare coverage to over 99% of the population in our country. Our centre specifically cares to MS patients within the contributive branch. Although the coverage for the different DMTs in our country is mandated to be equal regardless of the individual insurers, several aspects of the care of MS might differ across them. These include, for example, the access to continuous and specialised consultations, regular imaging monitoring and continuous DMT provision, among others, which might have influenced MS activity, and thus the need for treatment changes. The rather long persistence on treatments found in our study might also stem from coverage issues. Since healthcare resources are administered by the private insurers, and newer approved therapies enter the market usually at higher costs, the provision of the latter might have been withheld by the former. However, our study is unable to confirm this, and it is thus a matter of further investigation. Our results show that the insurer was determinant in the likelihood of switching treatment, but also the care under our specialised centre, although with effects in opposite directions. Our interpretations for this are that the characteristics of healthcare provision of the two insurers before directing it to our centre influenced the treatment changes, and the care at our reference centre increased its likelihood by improved surveillance of MS activity and adverse events.

Having said that, it is important to note that, since choosing the insurer is a voluntary decision of every person, the insurer status of the pwMS might have been an expression of unobserved variables such as socioeconomic status and educational attainment, which are known to be related to access barriers [24, 27], and might have influenced the treatment persistence as well.

We acknowledge several limitations of our study, the most important of which is its retrospective design, which introduces a high risk of recall bias. This is particularly important since most of the pwMS were already treated and several had treatment switches before being cared for at our institution. While the year of DMT onset and stopping was known in the totality of the cases, in only half of them we knew the month and the exact date in a minority. Also, the design of our study precludes the assessment of the relationship between disability at the onset and during the course and the treatment choice. There is a high risk of selection bias as well. pwMS with more aggressive MS or with more administrative difficulties in the past might have been drawn to seek care at our reference centre. This might be manifest in our results, as a high proportion of pwMS receiving treatment, and it is likely that patients with mild disease (whether under treatment or not) had not sought care in our specialised centre. Nevertheless, the disability in our sample is rather low, which might reflect the care for new onset and younger patients. Although limited by this, our findings might be generalizable to other urban populations in our country given the conditions of our healthcare system. However, roughly half of the population in our country is not covered by the contributive healthcare system, and nearly 20% of the population lives in rural areas. Therefore, our results might not be generalizable to that portion of the population.

## Conclusions

In conclusion, our findings suggest that a majority of people with MS will have treatment changes, most likely driven by disease activity, but strongly influenced by coverage and care-related factors. Most treatment changes have occurred involving interferons, likely due to being the first DMTs available. Further study is needed to assess persistence and its determinants in healthcare settings from other Latin-American countries.

### Supplementary Information

**Supplementary Material 1.**

## Data Availability

Anonymized data are available upon reasonable request with the ethics committee’s authorization. Requests for data should be done directly to the corresponding author.

## Abbreviations

ALZ: Alemtuzumab
DMF: Dimethylfumarate
DMTs: Disease-modifying therapies
EDSS: Expanded disability status score
EMA: European medicines agency
FDA: Food and drug administration
FTY: Fingolimod
GA: Glatiramer acetate
IFN: Interferons
JC: John Cunningham virus
MS: Multiple sclerosis
NTZ: Natalizumab
OCR: Ocrelizumab
pwMS: People with Multiple Sclerosis
RTX: Rituximab
SUS: Treatment suspension
TRF: Teriflunomide
