# Supplementary material for: Treatment patterns and persistence on disease modifying therapies for multiple sclerosis and its associated factors
Source: BMC Neurol. 2024 Apr 2;24:108. doi: 10.1186/s12883-024-03594-3 (PMC10986095; doi:10.1186/s12883-024-03594-3)
Supplement: Supplementary file 1 — Supplementary Material 1. [file 12883_2024_3594_MOESM1_ESM.docx]

**Supplementary table S1**. STROBE Statement- checklist

|  | **Item No.** | **Recommendation** | **Page  No.** | **Relevant text from manuscript** |
| --- | --- | --- | --- | --- |
| **Title and abstract** | 1 | (a) Indicate the study’s design with a commonly used term in the title or the abstract |  | We conducted a retrospective observational study including… |
|  |  | (b) Provide in the abstract an informative and balanced summary of what was done and what was found |  | We estimated the overall incidence rate of medication changes and assessed the persistence on medication with Kaplan-Meier survival estimates for individual medications and according to efficacy and mode of administration. The factors associated with changing medications were assessed using adjusted Cox proportional-hazards models. |
| **Introduction** | | | |  |
| Background/rationale | 2 | Explain the scientific background and rationale for the investigation being reported |  | DMTs impose an important economic burden on the healthcare systems, accounting for more than two-thirds of the total direct cost of MS care.. The economic impact of MS in the healthcare system of Colombia has been scarcely studied, and the behaviour of DMTs prescription is unknown. |
| Objectives | 3 | State specific objectives, including any prespecified hypotheses |  | … the aim of this study was to ascertain the persistence on DMTs, investigate the frequency, reasons and determinants for switching between DMTs, and describe their prescription patterns in a real-life scenario in our country. |
| **Methods** | | | |  |
| Study design | 4 | Present key elements of study design early in the paper |  | … observational study performed in a single centre… |
| Setting | 5 | Describe the setting, locations, and relevant dates, including periods of recruitment, exposure, follow-up, and data collection |  | We included all people with MS (pwMS) confirmed according to the 2017 revisions of the McDonald criteria, who had had at least one visit in our centre between May 2016 through December 2020 |
| Participants | 6 | Cross-sectional study—Give the eligibility criteria, and the sources and methods of selection of participants |  | We included all people with MS (pwMS) confirmed according to the 2017 revisions of the McDonald criteria[10], who had had…  Records were retrospectively reviewed between September and December 2020 and data were gathered using… |
|  |  | (b) Cohort study—For matched studies, give matching criteria and number of exposed and unexposed  Case-control study—For matched studies, give matching criteria and the number of controls per case |  | NA |
| Variables | 7 | Clearly define all outcomes, exposures, predictors, potential confounders, and effect modifiers. Give diagnostic criteria, if applicable |  | The main outcome was a change in the DMT used and was defined as the moment when a subsequent DMT was started… |
| Data sources/ measurement | 8* | For each variable of interest, give sources of data and details of methods of assessment (measurement). Describe comparability of assessment methods if there is more than one group |  | Records were retrospectively reviewed between September and December 2020 and data were gathered using Redcap[11]. Basic demographic (age, sex) as well as clinical variables such as age and MS phenotype at onset[12], disease duration, last disability assessment (using the expanded disability status scale EDSS[13]) and age at treatment onset were collected. For each DMT started, the date of the first and last dose was documented. If these dates were unknown, they were imputed according to a pre-specified protocol. |
| Bias | 9 | Describe any efforts to address potential sources of bias |  | As this is a retrospective study based on historical records it is subject to a strong recall bias. In order to mitigate it at least for the basic demographic and clinical variables, we have developed a standard registration format in our electronic medical records. |
| Study size | 10 | Explain how the study size was arrived at |  | We did not perform an a priori sample size calculation. Given that the necessary information was easily accessible, we aimed to assess the census of our cohort. For the |

| Quantitative variables | 11 | Explain how quantitative variables were handled in the analyses. If applicable, describe which groupings were chosen and why |  | Quantitative variables were described in terms of central tendency measures (mean/median) and dispersion measures (standard deviation [SD]/interquartile range [Q1-Q3]) according to their statistical distribution, which was assessed using Q-Q plots and the Shapiro-Wilks test. |
| --- | --- | --- | --- | --- |
| Statistical methods | 12 | (a) Describe all statistical methods, including those used to control for confounding |  | …we estimated the cumulative hazard over time using Kaplan-Meier estimates assuming the changes were recurrent in nature. …risk of treatment changes in general with hazard ratios (HR) calculated from multivariate models, using the Prentice, Williams and Peterson-gap time version of the Cox proportional hazard model. …The models included the reasons for each treatment change (classified as disease activity, safety and others), in order to assess if the baseline variables were independently associated with the risk of treatment switches. |
|  |  | (b) Describe any methods used to examine subgroups and interactions |  | Persistence on DMTs was also analysed by mode of administration and high vs. low efficacy |
|  |  | (c) Explain how missing data were addressed |  | NA |
|  |  | (d) Cross-sectional study—If applicable, describe analytical methods taking account of sampling strategy |  | NA |
|  |  | (e) Describe any sensitivity analyses |  | NA |
| **Results** | | | | |
| Participants | 13* | (a) Report numbers of individuals at each stage of study—eg numbers potentially eligible, examined for eligibility, confirmed eligible, included in the study, completing follow-up, and analysed |  |  |
|  |  | (b) Give reasons for non-participation at each stage |  |  |
|  |  | (c) Consider use of a flow diagram |  |  |
| Descriptive data | 14* | (a) Give characteristics of study participants (eg demographic, clinical, social) and information on exposures and potential confounders |  | Table 1 |
|  |  | (b) Indicate number of participants with missing data for each variable of interest |  | NA |
|  |  | (c) Cohort study—Summarise follow-up time (eg, average and total amount) |  | NA |
| Outcome data | 15* | Cohort study—Report numbers of outcome events or summary measures over time |  | NA |
|  |  | Case-control study—Report numbers in each exposure category, or summary measures of exposure |  | NA |
|  |  | Cross-sectional study—Report numbers of outcome events or summary measures |  | …a total of 710 treatment changes: 404 (56.9%) were switches between DMTs, 186 (26.2%) were treatment suspensions and 120 (16.9%) were treatment resumptions. |
| Main results | 16 | (a) Give unadjusted estimates and, if applicable, confounder-adjusted estimates and their precision (eg, 95% confidence interval). Make clear which confounders were adjusted for and why they were included |  | The overall incidence (95%CI) of any treatment change was 1.09 (1.01-1.17) per patient-years.  Table 3. Association of treatment changes with clinical and demographic variables according to univariable and multivariable Cox proportional hazard models. |
|  |  | (b) Report category boundaries when continuous variables were categorized |  | NA |
|  |  | (c) If relevant, consider translating estimates of relative risk into absolute risk for a meaningful time period |  |  |

| Other analyses | 17 | Report other analyses done—eg analyses of subgroups and interactions, and sensitivity analyses |  | The measures of DMT importance within the network showed that the most central DMTs were the interferons (eigenvector centrality: 1.0; betweenness: 0.0; closeness: 0.019), followed by the status of suspension (eigenvector centrality: 0.888; betweenness: 0.0; closeness: 0.016). The most marginal DMT was ocrelizumab (eigenvector centrality: 0.097) (Figure 3 and Supplementary Table S3) |
| --- | --- | --- | --- | --- |
| **Discussion** | | | | |
| Key results | 18 | Summarise key results with reference to study objectives |  | …most pwMS who started treatment had a DMT switch at some point, being disease activity the most common reason for change and that the persistence on treatment was similar for the different DMTs, regardless of their efficacy or mode of administration. In addition, the insurer status of the pwMS was found to be the main determinant of DMT switching. Most treatment switches involved interferons fingolimod and treatment suspension. |
| Limitations | 19 | Discuss limitations of the study, taking into account sources of potential bias or imprecision. Discuss both direction and magnitude of any potential bias |  | We acknowledge several limitations of our study, the most important of which is its retrospective design, which introduces a high risk of recall bias. This is particularly important due… |
| Interpretation | 20 | Give a cautious overall interpretation of results considering objectives, limitations, multiplicity of analyses, results from similar studies, and other relevant evidence |  | In our study, age at MS onset and insurer status were the only variables associated with the risk of switching therapies. The former has several interpretations. It is possible that pwMS with younger age at onset had a more active disease than pwMS with onset at an older age, which might increase the likelihood of treatment switching due to lack of efficacy… |
| Generalisability | 21 | Discuss the generalisability (external validity) of the study results |  | Although limited by this, our findings might be generalizable to other urban populations in our country (given the conditions of our healthcare system)... |
| **Other information** | |  | | |
| Funding | 22 | Give the source of funding and the role of the funders for the present study and, if applicable, for the original study on which the present article is based |  | This study was supported by a grant from Biogen-Idec, no CO-MSG-11811, with no influence on the design, execution or statistical analysis from the sponsor. |

**Supplementary Figure S1:** Kaplan-Meier survival curves of persistence on each individual DMD. RT: Rituximab; ALZ: Alemtuzumab; FTY: Fingolimod; DMF: Dimethylfumarate; IFN: Interferons; OCR: Ocrelizumab; NTZ: Natalizumab; TF: Teriflunomide; COP: Glatiramer acetate.


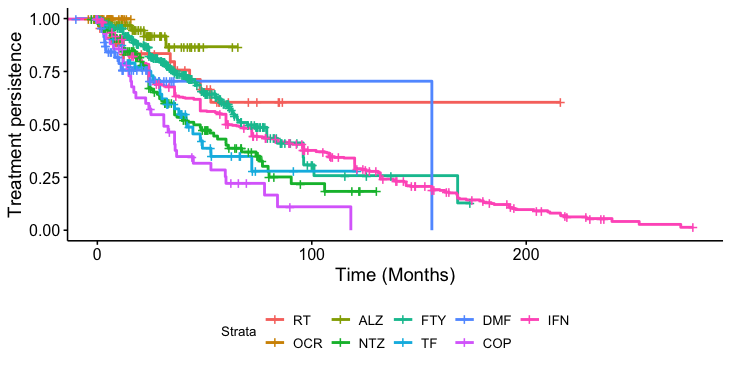


**Supplementary Table S2:** Schoenfeld’s test for the proportional hazards assumption of the multivariable cox model.

| **Variable** | | **Chi-squared** | **p-value** |
| --- | --- | --- | --- |
| HUN | | 19.115 | 1.2e-05 |
| Age at MS onset | | 0.876 | 0.349 |
| Diagnostic delay | | 5.257 | 0.022 |
| Disease duration | | 32.191 | 1.4e-08 |
| Sex | | 0.890 | 0.345 |
| Insurer | | 0.518 | 0.472 |
| Phenotype | | 1.574 | 0.210 |
| Reason for treatment switch | Disease activity | 3.768 | 0.052 |
|  | Safety | 5.860 | 0.015 |
|  | Others | 2.210 | 0.137 |
| Global | | 76.634 | 2.3e-12 |

**Supplementary table S3**. Treatment switches matrix

|  |  | **Switching to** | | | | | | | | | |
| --- | --- | --- | --- | --- | --- | --- | --- | --- | --- | --- | --- |
|  |  | **Alemtuzu-mab** | **Glatiramer** | **Dimethylf-umarate** | **Fingolimod** | **Interferons** | **Natalizum-ab** | **Ocrelizum-ab** | **Rituximab** | **Suspensi-on** | **Teriflunom-ide** |
| **Switching from** | **Alemtuzumab** | – | 0 (0.0) | 0 (0.0) | 0 (0.0) | 0 (0.0) | 1 (20.0) | 1 (20.0) | 1 (20.0) | 2 (40.0) | 0 (0.0) |
|  | **Glatiramer** | 0 (0.0) | – | 2 (6.1) | 9 (27.3) | 5 (15.2) | 6 (18.2) | 0 (0.0) | 0 (0.0) | 11 (33.3) | 0 (0.0) |
|  | **Dimethylfumarate** | 2 (16.7) | 0 (0.0) | – | 1 (8.3) | 0 (0.0) | 0 (0.0) | 1 (8.3) | 0 (0.0) | 5 (41.7) | 3 (25.0) |
|  | **Fingolimod** | 26 (25.5) | 2 (2.0) | 2 (2.0) | – | 2 (2.0) | 19 (18.6) | 15 (14.7) | 13 (12.7) | 21 (20.6) | 0 (0.0) |
|  | **Interferons** | 4 (1.2) | 13 (4.0) | 11 (3.3) | 104 (31.6) | – | 52 (15.8) | 0 (0.0) | 7 (2.1) | 106 (32.2) | 28 (8.5) |
|  | **Natalizumab** | 9 (12.7) | 0 (0.0) | 0 (0.0) | 23 (32.4) | 0 (0.0) | – | 7 (9.9) | 7 (9.9) | 24 (33.8) | 1 (1.4) |
|  | **Ocrelizumab** | 0 (0.0) | 0 (0.0) | 0 (0.0) | 0 (0.0) | 0 (0.0) | 0 (0.0) | – | 0 (0.0) | 0 (0.0) | 0 (0.0) |
|  | **Rituximab** | 1 (7.7) | 0 (0.0) | 0 (0.0) | 1 (7.7) | 0 (0.0) | 1 (7.7) | 1 (7.7) | – | 9 (69.2) | 0 (0.0) |
|  | **Suspension** | 6 (4.8) | 6 (4.8) | 10 (8.1) | 41 (33.1) | 12 (9.7) | 23 (18.5) | 6 (4.8) | 9 (7.3) | – | 8 (6.5) |
|  | **Teriflunomide** | 2 (6.5) | 0 (0.0) | 1 (3.2) | 13 (41.9) | 1 (3.2) | 3 (9.7) | 3 (9.7) | 0 (0.0) | 8 (25.8) | – |

**Supplementary table S4.** Centrality measures within the DMDs network

| **DMD** | **In-degree** | **Out-degree** | **Betweenness** | **Eigenvector centrality** | **Closeness** |
| --- | --- | --- | --- | --- | --- |
| **Fingolimod** | 7 | 8 | 8.5 | 0.839 | 0.029 |
| **Natalizumab** | 7 | 6 | 8.0 | 0.570 | 0.034 |
| **Interferons** | 4 | 8 | 0.0 | 1.000 | 0.019 |
| **Glatiramer** | 3 | 5 | 1.5 | 0.187 | 0.025 |
| **Suspension** | 8 | 9 | 0.0 | 0.888 | 0.016 |
| **Teriflunomide** | 4 | 7 | 13.0 | 0.238 | 0.043 |
| **Dimethylfumarate** | 5 | 5 | 20.0 | 0.120 | 0.042 |
| **Rituximab** | 5 | 5 | 4.5 | 0.164 | 0.056 |
| **Alemtuzumab** | 7 | 4 | 26.0 | 0.168 | 0.053 |
| **Ocrelizumab** | 7 | 0 | 0.0 | 0.097 | 0.011 |
